# A New Method of Brushing Teeth

**Published:** 1916-07

**Authors:** Henry Barnes

**Affiliations:** Cleveland


### A NEW METHOD OF BRUSHING TEETH.

BY HENRY BARNES, D.D.S. CLEVELAND.

This method has for its object the cleansing of the interproximal spaces as well as the labial and lingual surfaces. In the main this is accomplished by the use of a specially designed brush and without the accompaniment of abrasive or detergent tooth toilet agents other than water alone. The method of using the brush has much to do with the efficiency of the brush and water. The following extract from the Cleveland *News* of June 3, 1916, and extracts from a paper read before the Northern Ohio Dental Association will serve to call attention to the method. The paper in full will be printed with illustrations in the *Dental Summary.*

"From childhood up men and women now of mature years have had it impressed upon their minds that healthy teeth are essential to healthy bodies; that unremitting care of the teeth is demanded by a sane regard for life and happiness as well as by due consideration of cleanliness and appearance, and that correct care of the teeth consists principally of brushing them after meals with regularity and vigor, to say nothing of stiff bristles and saponaceous powder, paste or liquid.

"Have those who have been pursuing this course religiously for many years been ruining their teeth rather than preserving them? In their sedulous efforts to have good teeth have they been assuring themselves worse teeth than are possessed by persons who never made any such effort? The question's importance is obvious, if teeth are as precious as we have been led to believe and if mistaken prudence is imperiling what it is intended to preserve.

"That Americans are in the pernicious toils of just such an error has been not only suggested but asserted, before the Northern Ohio Dental Association, in a notable paper on 'Conservation of Gum and Tooth Tissue,' prepared by Dr. Henry Barnes, Cleveland veteran of the dental profession. His contention, based on wide observation and experiment, is, in brief, that the prevalence of various ills of tooth and gum preliminary to and including pyorrhea is due in large measure to prevailing tooth brushing practices and that these maladies can be relieved and prevented by reforming those practices.

"Instead of using brushes consisting of several rows of stiff bristles and brushing energetically with vertical, lateral or circular motions, Dr. Barnes recommends the use of a brush containing only one row of softer and finer bristles, applying it always in the direction away from the gums and toward the edge of the teeth, employing the sides rather than the ends of the bristles in a sliding stroke, after the manner of shaving, and substituting gentleness and patience for vigor at all times.

"It seems but fair to aid in spreading knowledge of a theory which must impress even the lay mind as plausible, when ignorance of it can have consequences so far-reaching. It would seem the duty of the dentists to lose no time in proving it correct or otherwise—and in informing the public. If TOOTH DESTRUCTION IS BEING PRACTICED AND TAUGHT IN THE GUISE OF TOOTH CONSERVATION, the people can not be told the truth TOO SOON, and the telling will be a LAUDABLE PUBLIC SERVICE."

The brush itself is made small and very narrow. It has but one row of bristles, which are of the finer character and are set in small bunches. Each alternating bunch is cut low so as to give the surface of the bristles a serrated form, which will permit the bristles passing readily into the proximal spaces and all irregularities of the teeth. It may be called a pick brush because of its form and the method of using.

The method of holding and using is like that of holding and using a razor in shaving. The brush is held lightly in the hand as a razor or pen would be held, and instead of using an up-and-down or rotary motion, as is usually advocated, the brush is applied with the ends of the bristles directed from the gum over the roots of the teeth toward the occlusal surfaces. The direction of the brushing is vertical or in line with the axis of the tooth. The motion should not be made by rotating the wrist, but by a sweeping motion by a shoulder and arm movement. In this way the ends of the bristles, in making the positive motion, pass into the proximal spaces and carry the debris before the brush into the oral space, and the return motion exercises a gentle massaging of the gum tissues.

The brush is held limply in the right hand, with the tips of the fingers—the thumb and forefinger holding the handle close to the bristles, the first, second and third fingers on the back of the brush, the thumb and little finger underneath.

The thumb and forefinger act as the pivot about which the brush rotates as it moves around the arch to accommodate its fullest action to the curves and surfaces of the dental arch.

The second and third and little fingers act as the motors which move the brush handle, maintaining it parallel to the plane of occlusion as the brush is moved around the arch.

Relax all muscles and carefully insert the brush into the mouth, carrying it back to the last right upper molar region without touching the teeth or gums until the brush is laid high upon the gums, the bristles pointing vertically downward. Keep the side of the back of the brush as close to the gums as is possible without touching them and lightly draw the brush vertically downward over the gums and teeth. This forms the working stroke which cleans and gently massages the gums, including the gum septa.

This action squeezes the gingivae and gum septa, forcing debris out of the gingival margins and inaccessible portions of the interproximal spaces onto tooth surfaces and into the accessible interproximal spaces where the short and long bristle ends scrape, shave or scoop the debris up and carry it away. During brushing it is highly important that the brush be frequently rinsed in running water—warm preferred.

The return stroke is inert except for a gentle gum massage, the bristles sliding diagonally forward from the occlusal margin over the tooth and gum towards the root apices. This action places the brush high on the gums in position for the next vertical stroke. The vertical stroke and diagonal return strokes are repeated again and again with frequent brush rinsing until the bristles reach as far as the left upper cuspid.

Then the brush is removed from the mouth and reversed, the bristle ends now pointing upward. The finger hold the same as before. The brush is now in position for action on the lower jaw, from molars to left cuspid. The motion now is vertically upward for cleaning and massaging and obliquely forward and downward for the practically inert return stroke.

For the left side of the mouth, from left molars to right cuspid, the brush may be held in the left hand in the same manner as for the right side, but since few people are ambidextrous, the following method is advised:

The brush is taken in the hand limply as a pen or pencil should be held between the thumb and first and second fingers of right hand. The thumb is next to bristles which point downward. Place the brush on gums in left upper molar region in the same manner as it was placed on the right side. The action is the same as that used for the right side to right cuspid.

For lingual brushing, the bristles are laid lengthwise upon the gums over the apical region and drawn or shaved over the gums and teeth to the occlusal surfaces. The lingual brush has soft bristles parallel to the handle which practicaly compels its correct use. The only incorrect strokes of this brush would be mesially or distally oblique to the longitudinal axes of the teeth, or where the brush is held rigidly or used too vigorously. In lingual brushing about the curve of the arch and beyond, the brush itself must be rotated in the fingers to compensate for the curves of the arch.

In all brushing, the bristles act as a gum and tooth cleanser and as a gum massage when the gentle working stroke is from the region of the apices to the occlusal surfaces. In the return stroke the bristles do not lose contact with the tissues—they slide back mesio-obliquely to the apical position without any other action than that of a gentle gum massage.

